# Off-pump Versus On-pump Coronary Artery Bypass Grafting in Diabetic patients: A Meta-analysis of Observational Studies with a Propensity-Score Analysis

**DOI:** 10.1007/s10557-024-07603-y

**Published:** 2024-07-11

**Authors:** Qiushi Ren, Gang Li, Tongxin Chu, Quan Liu, Yang Huang, KaiZheng Liu, Jinyu Pan, Zhongkai Wu

**Affiliations:** 1 Department of Cardiac Surgery, First Affiliated Hospital of Sun Yat-Sen University, Guangzhou, China; 2 NHC Key Laboratory of Assisted Circulation, Sun Yat-Sen University, Guangzhou, China

**Keywords:** Coronary artery bypass grafting, On-pump, Off-pump, Diabetes, Mortality, Cerebrovascular accident

## Abstract

**Purpose:**

The debate between off-pump coronary artery bypass grafting (OPCAB) and on-pump coronary artery bypass grafting (ONCAB) in diabetic patients remains. This meta-analysis aimed to investigate outcomes after OPCAB versus ONCAB for patients with diabetes.

**Methods:**

Literature research was conducted up to December 2023 using Ovid Medline, EMBASE, and the Cochrane Library. Eligible studies were observational studies with a propensity-score analysis of OPCAB versus ONCAB. The primary outcomes were early mortality and mid-term survival. The secondary outcomes were cerebrovascular accidents, reoperation for bleeding, incomplete revascularization, myocardial infarction, low cardiac output, and renal replacement therapy.

**Results:**

Our research identified seven observational studies with a propensity-score analysis enrolling 13,085 patients. There was no significant difference between OPCAB and ONCAB for early mortality, mid-term survival, myocardial infarction, low cardiac output, and renal replacement therapy. OPCAB was associated with a lower risk of cerebrovascular accidents (OR 0.43; 95% CI, 0.24–0.76, *P* = 0.004) and reoperation for bleeding (OR 0.60; 95% CI, 0.41–0.88, *P* = 0.009). However, OPCAB was associated with a higher risk of incomplete revascularization (OR 2.07; 95% CI, 1.60–2.68, *P* < 0.00001).

**Conclusion:**

Among patients with diabetes, no difference in early mortality and mid-term survival was observed. However, OPCAB was associated with a lower incidence of morbidity, including cerebrovascular accidents and reoperation for bleeding.

**Supplementary Information:**

The online version contains supplementary material available at 10.1007/s10557-024-07603-y.

## Introduction

By 2030, the number of people with type 2 diabetes worldwide is expected to reach 7079 per 100,000 [1]. The increasing prevalence of type 2 diabetes is a global health concern. The atherosclerotic cardiovascular disease is the primary cause of death for individuals with diabetes [2]. About 25 to 40% of patients undergoing myocardial revascularization have diabetes [3]. They are a high-risk group for coronary artery bypass surgery (CABG) and percutaneous coronary intervention (PCI), who are more likely to suffer from complex coronary heart disease and have a higher incidence of postoperative adverse events than non-diabetic patients [4].

In patients with multi-vessel coronary artery disease and diabetes, CABG had a mortality benefit over PCI [5]. However, the debate between OPCAB and ONCAB has lasted over three decades [6]. OPCAB can avoid the use of cardiopulmonary bypass and reduce aortic manipulations, but the quality of coronary revascularization has been questioned. Previous studies showed that OPCAB has benefits in high-risk patients, including elderly patients and patients with low ejection fraction, but which type of CABG has a better prognosis in diabetic patients remains unclear. Many randomized controlled trials compared OPCAB and ONCAB in unselected patients. Still, few randomized controlled trials (RCT) have been conducted in patients with diabetes. Although two studies [7, 8] from the ROOBY trial reported early and mid-term results after OPCAB and ONCAB in diabetic patients, they were both post hoc analyses, and patients’ allocation conversion may influence the outcomes. Some observational studies comparing the two surgical techniques in diabetic patients have unbalanced preoperative baseline data, which reflected patient selection biases. When results from these non-randomized studies are included in meta-analyses, they should always be evaluated cautiously. A propensity-score analysis, including matching and covariate adjustment, is a powerful tool to strengthen causal inferences drawn from observational studies [9].

To determine whether OPCAB improves outcomes in diabetic patients compared with ONCAB, we performed a meta-analysis of observational studies using propensity-score analysis.

## Methods

### Search Strategy

This systematic review was conducted according to the Preferred Reporting Items for Systematic Reviews and Meta-Analyses (PRISMA) 2020 and AMSTAR (Assessing the methodological quality of systematic reviews) Guidelines [10, 11]. The study was preregistered at the International Prospective Register of Systematic Reviews (PROSPERO). We systematically searched the major databases through December 17, 2023, including Ovid MEDLINE, EMBASE, and the Cochrane Library, using keywords and Medical Subject Headings (MeSH). The search strategy included the following terms: “off-pump,” “Coronary Artery Bypass,” “CABG,” “coronary artery bypass graft,” and “Diabetes Mellitus”). (details about the complete search strategy are available in Supplemental Table 1).

**Table 1:** Characteristics of the included studies

Study	Observation period	Study location	Study centers (no.)	Design		Patient OPCAB versus ONCAB	
				Type	Detail	Crude	Analysis
Srinivasan (2004)	04/1997–09/2002	United Kingdom	1	Covariate adjustment	Logistic regression	186,765	186,765
Emmert (2010)	2002–2008	Switzerland	1	Covariate adjustment	Logistic regression	540,475	540,475
Renner (2013)	02/2009–10/2011	Germany	1	Covariate adjustment	Logistic and Cox regression	355,502	355,502
Singh (2016)	01/2001–03/2005	USA, Canada, Brazil, Mexico, the Czech Republic and Austria	49	Matching	0.01 caliper	444,171	153,153
Benedetto (2016)	04/1996–04/2015	United Kingdom	1	Matching	0.2 caliper	1253,1197	995,995
Huang (2019)	2000–2011	China (Taiwan)	Unknown	Matching	0.2 caliper	4104,9405	3796,3796
Song (2023)	01/2011–01/2021	China	1	Matching	Nearest neighbor	352,196	187,187

### Inclusion and Exclusion Criteria

The studies were screened by two authors independently, and any discrepancies regarding a study’s eligibility were resolved by discussion and consensus. The inclusion criteria were as follows: (1) studies comparing OPCAB versus ONCAB in diabetic patients; (2) studies using propensity-score analysis; (3) studies published with full available text in English. The exclusion criteria were as follows: (1) concomitant surgical interventions other than CABG; (2) reviews, letters, editorials, conference abstracts, and case reports.

### Data Extraction and Quality Assessment

All data were extracted independently and crosschecked by two review authors. Any disagreements between the two authors were discussed until they reached a consensus. Disagreements were resolved by discussion with a third author when necessary. The following variables were included: study demographics (the lead author, publication year, observation period, study location, number of centers, design, and sample size); patient demographics; comorbidities (age, sex, hypertension, myocardial infarction history, ejection fraction, renal dysfunction); and outcomes. In case of discrepancies, they were resolved by consultation. The quality of the included papers was assessed using the Newcastle–Ottawa Scale [12].

### Outcomes

The primary outcomes were early mortality and mid-term survival. Early mortality was defined as any cause of death within 30 days after surgery or before discharge. Mid-term was defined as a follow-up of at least more than 1 year. Secondary outcomes included the following: (1) cerebrovascular accident; (2) bleeding complications; (3) incomplete revascularization; (4) myocardial infarction; (5) renal replacement therapy; (6) low cardiac output.

### Statistical Analysis

All statistical analysis was conducted using RevMan 5.3 software or R software. Random-effects models with inverse variance and Mantel–Haenszel were chosen, and odds ratios (OR) and hazard ratios (HR) with 95% confidence intervals (CIs) and *P* values (considered statistically significant when *P* < 0.05) were calculated and used to represent the results following analysis. The heterogeneity was evaluated by the chi[2] test and calculated by *I*^2^ statistic. Clinical outcomes were presented through the graphic presentation in Forest plots. Publication bias was also assessed through funnel plots. Subgroup analysis of the primary outcomes was performed according to the propensity score method (propensity score matching and propensity score covariate adjustment) adopted by the included studies.

## Results

### Study Characteristics

**Fig. 1:**
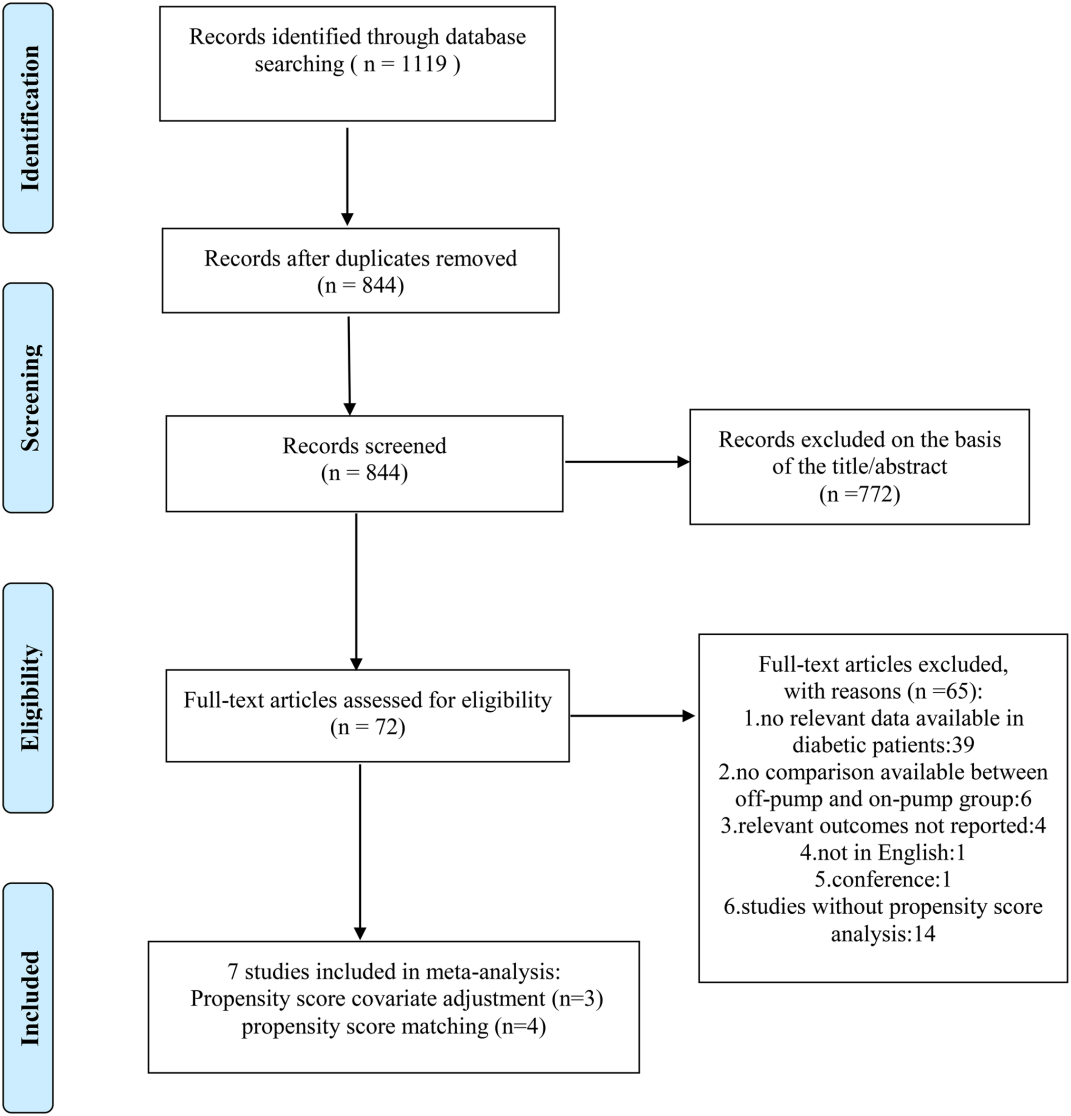
PRISMA flow diagram

A total of 1119 records were identified through an initial search, and 7 trials [13–19] published between 2004 and 2023 were finally included (Fig. 1). Among the included studies, four used propensity-score matching, and three used propensity-score covariate adjustment. Overall, 6212 patients were treated with OPCAB, whereas 6873 were treated with ONCAB.

The main characteristics of the included trials are shown in Table 1, and the baseline characteristics of the patients are summarized in Table 2. The mean age of the OPCAB participants varied from 59.9 to 70.0 years, whereas the mean age of the ONCAB participants ranged from 60.4 to 69.0 years. The percentage of female participants, hypertension, myocardial infarction history, renal disease, and the mean number of grafts are also shown in Table 2. The risk of bias assessment is shown in Supplemental Table 2.

**Table 2:** Baseline characteristics of patients in the included studies

Study	OPCAB versus ONCAB							
	Mean age	Female, %	Hypertension, %	MI history, %	Renal disease, %	Mean LVEF, %	Insulin, %	Mean number of grafts
Srinivasan 2004	65.4, 65.2	22.6, 23.1	63.4, 62.7	NR	5.9, 4.2	< 30%, 11.3, 12.0	32.8, 25.7	3, 4*
Emmert 2010	65, 64	27, 23	58.5, 75.4	57.2, 65.3	5.7, 3.6	55, 56	NR	3.37, 3.83
Renner 2013	70, 69	21.1, 27.5	90.7, 92.2	34.6, 40.6	NR	60, 55	NR	NR
Singh 2016	62, 64	33, 27	49, 44	44, 34	NR	60, 59	NR	NR
Benedetto 2016	60–69, 60–69*	23.1, 23.4	NR	22.8, 20.0	3.9, 3.7	< 30%, 6.9, 7.4	41.4, 41.3	2.7, 3.0
Huang 2019	65.35, 65.19	29.95, 29.85	83.14, 83.69	28.85, 26.74	5.98, 5.92	NR	12.67, 13.41	NR
Song 2023	59.9, 60.4	35.3, 36.9	87.7, 87.7	34.8, 34.8	6.4, 7.0	60.2, 60.4	NR	NR

*MI* myocardial infarction, *LVEF* left ventricular ejection fraction, *NR* not reported

^*^Median

### Primary Outcomes

**Fig. 2:**
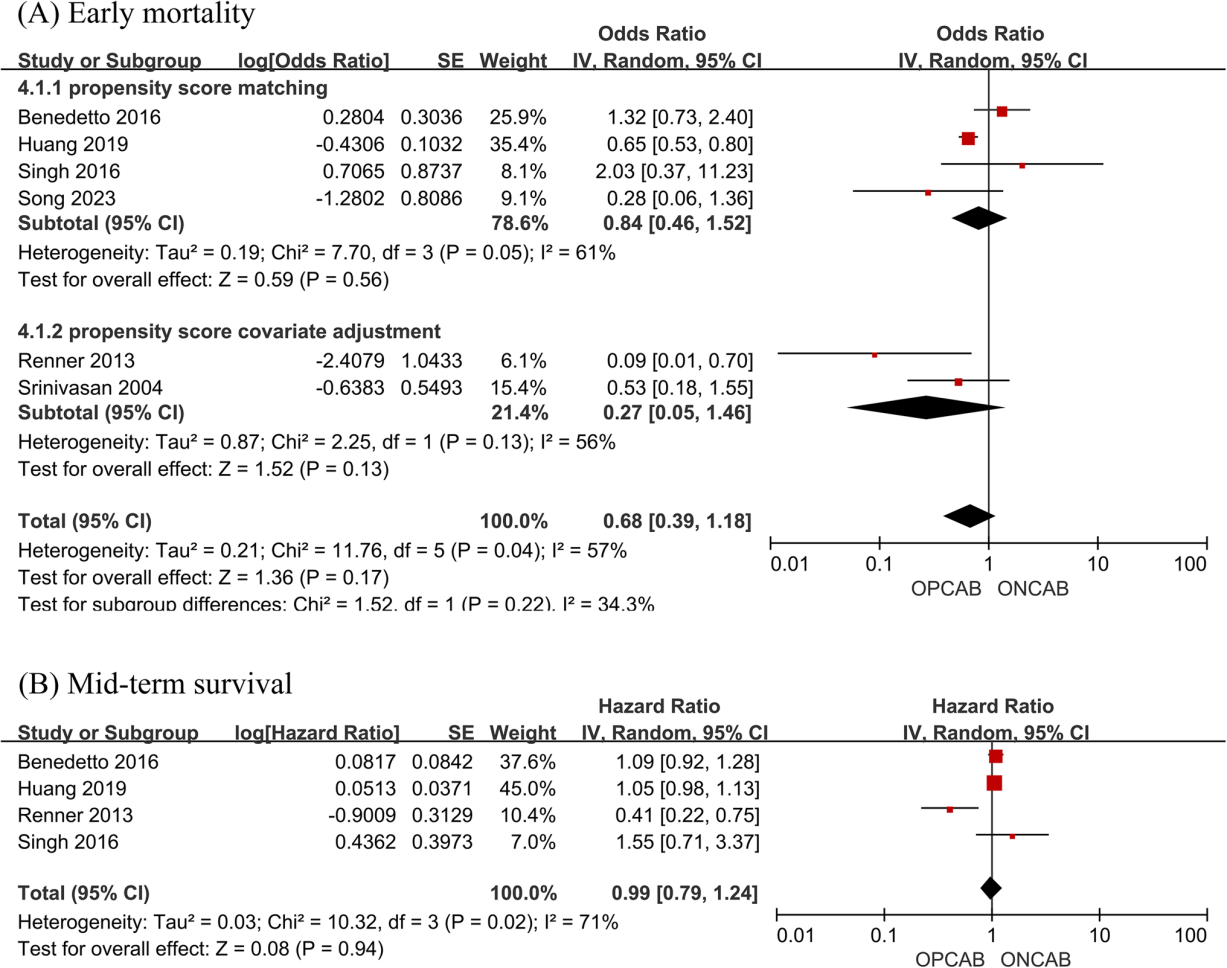
Forest plot for early mortality (**A**) and mid-term survival (**B**)

Except for one study without a definite follow-up period, six studies were included to compare the early mortality of patients with OPCAB versus ONCAB. There was no statistically significant difference in early mortality for OPCAB versus ONCAB (OR 0.68; 95% CI, 0.39–1.18, *P* = 0.17, *I*^2^ = 57%) among studies analyzed in propensity score matching (OR 0.84; 95% CI, 0.46–1.52, *P* = 0.56, *I*^2^ = 61%) and propensity score covariate adjustment (OR 0.27; 95% CI, 0.05–1.46, *P* = 0.13, *I*^2^ = 56%) (Fig. 2A).

Data from four studies with a more than 1-year follow-up also showed no difference in mid-term survival for OPCAB versus ONCAB (HR 0.99; 95% CI, 0.79–1.24, *P* = 0.94, *I*^2^ = 71%) (Fig. 2B).

### Secondary Outcomes

**Fig. 3:**
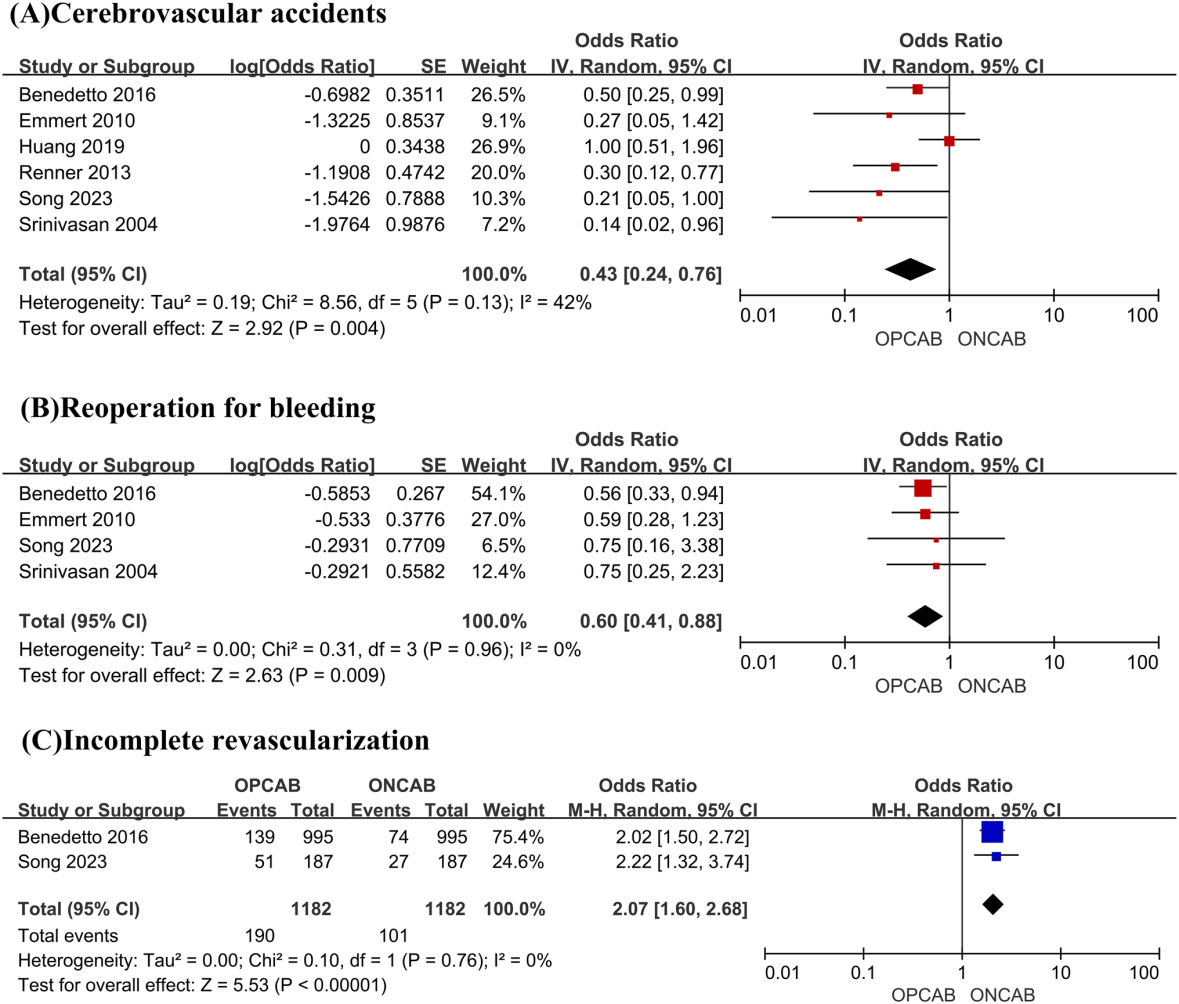
Forest plot for cerebrovascular accidents (**A**), reoperation for bleeding (**B**), and incomplete revascularization (**C**)

Compared with the ONCAB group, OPCAB was associated with a lower risk of cerebrovascular accidents (OR 0.43; 95% CI, 0.24–0.76, *P* = 0.004, *I*^2^ = 0%) (Fig. 3A) and reoperation for bleeding (OR 0.60; 95% CI, 0.41–0.88, *P* = 0.009, *I*^2^ = 0%) (Fig. 3B). Pooled data from two propensity score matching showed OPCAB was associated with a higher risk of incomplete revascularization (OR 2.07; 95% CI, 1.60–2.68, *P* < 0.00001) (Fig. 3C).

**Fig. 4:**
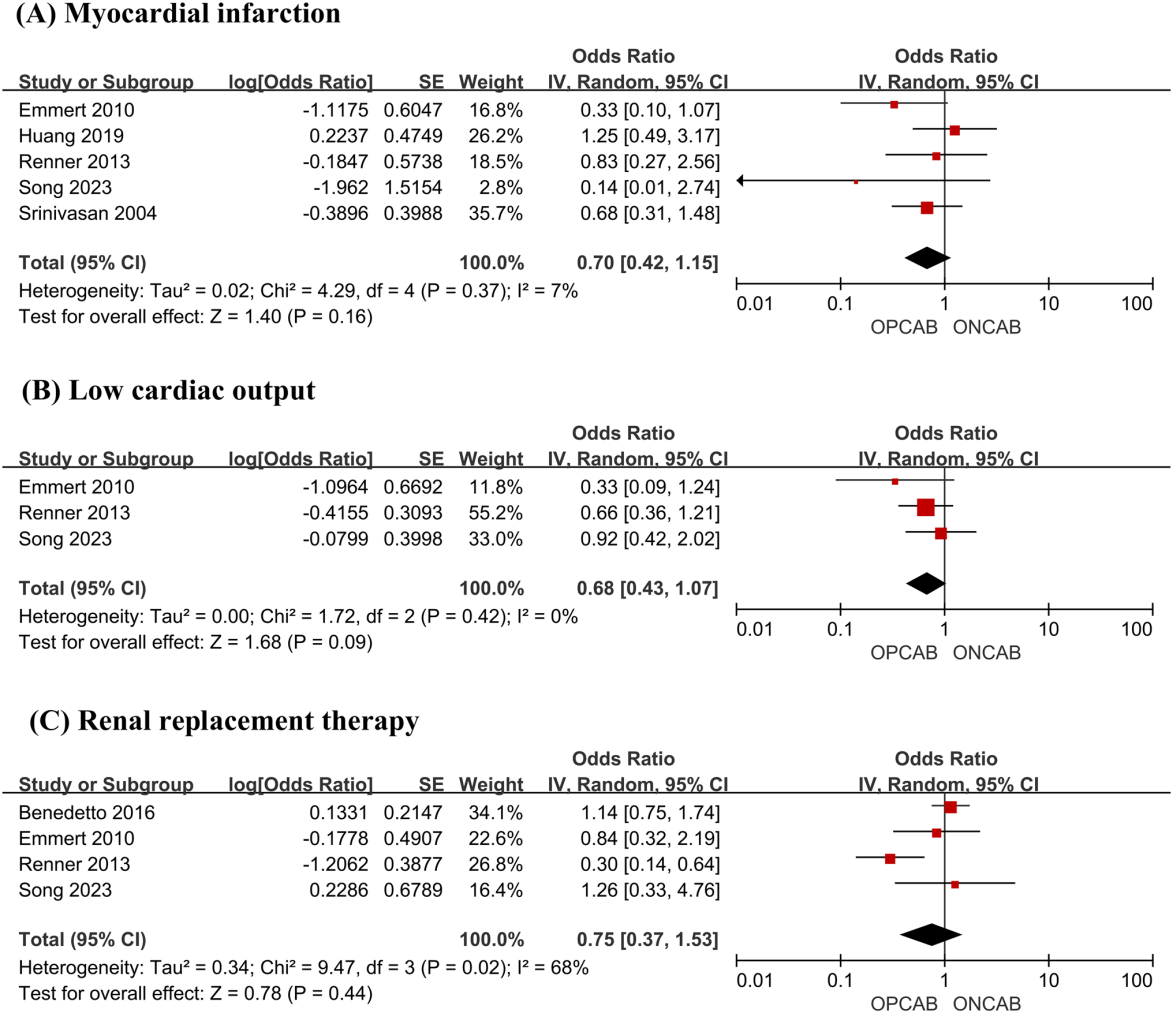
Forest plot for myocardial infarction (**A**), low cardiac output (**B**), and renal replacement therapy (**C**)

There was no statistically significant difference in myocardial infarction (OR 0.70; 95% CI, 0.42–1.15, *P* = 0.16, *I*^2^ = 7%) (Fig. 4A); low cardiac output for OPCAB versus ONCAB (OR 0.68; 95% CI, 0.43–1.07, *P* = 0.09, *I*^2^ = 0%) (Fig. 4B); and renal replacement therapy (OR 0.75; 95% CI, 0.37–1.53, *P* = 0.44, *I*^2^ = 68%) (Fig. 4C).

### Publication *Bias*

Only seven studies were included, so assessing them for publication bias seemed meaningless. Nevertheless, funnel plots were performed to show that studies presented a symmetric distribution, which indicated no obvious publication bias existed (Supplemental Fig. 1 and Fig. 2).

## Discussion

In this meta-analysis of observational studies with a propensity-score analysis, we explored outcomes after OPCAB or ONCAB for patients with diabetes mellitus. The main findings can be summarized as follows: (1) there was no significant difference in early mortality and mid-term survival. (2) OPCAB was associated with a lower risk of cerebrovascular accident and reoperation for bleeding but with a higher risk of incomplete revascularization.

A previous meta-analysis reported that OPCAB was associated with superior early mortality in high-risk groups, including octogenarians, patients with left ventricular dysfunction, and patients with chronic kidney disease [20–22]. However, our result showed that OPCAB and ONCAB had similar early mortality in patients with diabetes. Of note, one study [14] did not mention follow-up time for death and was thus not included in the analysis. Sensitivity analysis was performed by including the data in the study and reanalyzing it, and the difference still did not reach statistical significance (*P* = 0.07).

Our study revealed that there was no difference in mid-term survival for OPCAB versus ONCAB. A previous meta-analysis, including 7 RCTs with more than 4 years of follow-up, showed that compared with OPCAB in unselected patients, ONCAB appeared to offer superior long-term survival [23]. However, the evidence was deemed unsuitable due to using OR instead of HR, between-study heterogeneity, and fixed-effect model instead of random-effect model [24, 25]. Our study used HR and a random-effect model to overcome these disadvantages. Taggart et al. [26] found that off-pump and on-pump techniques achieved comparable long-term outcomes. However, OPCAB performed by low-volume off-pump surgeons was associated with a lower number of grafts and increased cardiovascular death. Completeness of revascularization and graft patency are the two factors contributing to the outcomes of CABG [23, 27]. Our result showed that OPCAB was associated with a higher risk of incomplete revascularization. In a more extended follow-up period, it may lead to lower survival [28]. With the development of heart positioning devices and the enrichment of surgeons’ experience, complete revascularization can be achieved in OPCAB [29].

OPCAB achieves better in postoperative morbidities, including cerebrovascular accident and reoperation for bleeding. Several previous studies demonstrated that compared with ONCAB, the incidence of stroke after OPCAB was lower [29, 30]. OPCAB eliminates the need for aortic cannulation and cross-clamping [31]. Lorusso et al. [30] showed a strong association between aortic manipulation and neurological outcomes after CABG surgery. The retrospective, multicenter, international study included 25,388 patients and suggested that the majority of the published research on the relationship between postoperative stroke and aortic manipulation was likely to be underpowered due to the low incidence of stroke and limited sample size. Considering that diabetic patients have a higher incidence of stroke after myocardial reperfusion therapy [32], OPCAB may provide additional benefits for this particular population.

Our study showed that OPCAB reduced the risk of reoperation for bleeding after surgery. Reduction of platelet counts and platelet dysfunction was a cause of bleeding after cardiopulmonary bypass [33, 34]. In addition, OPCAB does not require the establishment of extracorporeal circulation, and fewer incisions and sutures mean a reduced risk of bleeding [35]. Previous studies in other high-risk patients also reported higher blood transfusion rates in the ONCAB group [21, 22].

A recent study showed that the elevation of postoperative cardiac biomarkers (creatine kinase-MB and troponin I) after ONCAB was significantly higher than after OPCAB. However, they were quite lower than the threshold values defining postoperative myocardial infarction [36], which might explain the lack of differences in myocardial infarction in this analysis. Our study showed no difference in low cardiac output syndrome for OPCAB versus ONCAB. In contrast to our findings, previous meta-analyses suggested that OPCAB was associated with a lower incidence of low cardiac output in both the general and the re-operative population [37, 38]. More research is needed to confirm the result.

Our study did not show a significant difference in renal replacement therapy for OPCAB versus ONCAB. Similarly, a previous meta-analysis found no difference in renal failure between the two techniques in diabetic patients [39]. This meta-analysis included three more studies [17, 40, 41] than ours, and two [17, 41] had worse preoperative renal function in the OPCAB group. Our study only included studies using propensity-score matching or covariate adjustment, which could effectively reduce confounding bias. Of note, previous studies showed that in patients with preoperative moderate or severe renal failure, OPCAB technology has a protective effect on early postoperative outcomes, including the need for dialysis [42, 43]. Still, the protective effect was not observed in patients with mildly reduced renal function [43]. Thus, preoperative renal function may influence the protective effect of OPCAB, which needs more randomized controlled trials to verify.

This meta-analysis included seven observational studies that used the propensity-score analysis. Compared with a single small sample study, the meta-analysis had better statistical power. The current study suggested that in diabetic patients who need to undergo CABG, the patient’s perioperative risks and long-term outcomes should be comprehensively evaluated when choosing a surgical plan. The doctor’s OPCAB experience and the cost of treatment should also be considered.

### Limitation

Firstly, surgeon experience is vital for OPCAB. Since the number of surgeries performed by the surgeon was not provided in the included studies, subgroup analysis cannot be performed. Secondly, when comparing the mid-term mortality of OPCAB and ONCAB, data were from four studies due to the limited number of studies reporting long-term follow-up results. Despite the small number of studies, the results are still well-powered, considering that all included studies used a propensity-score analysis and the heterogeneity was low. We look forward to more studies with long-term follow-ups being published in the future. In addition, the subgroup analysis based on statistical design was not performed for mid-term survival because of the inclusion of only one propensity-score covariate adjustment study. Finally, due to the lack of grouping of preoperative renal function, subgroup analysis could not be performed to clarify whether preoperative renal function is related to the protective effect of OPCAB in diabetic patients.

## Conclusion

OPCAB provides significant benefits in terms of early postoperative complications, including cerebrovascular events and reoperation for bleeding. Although OPCAB and ONCAB were comparable in terms of early mortality and mid-term survival, ONCAB was associated with a lower risk for incomplete revascularization. Our findings can provide a scientific rationale for planning the appropriate operative strategy for future CABG cases with diabetes.

## Supplementary Information

Below is the link to the electronic supplementary material.

Supplementary file1 (DOCX 16.5 KB)

Supplementary file2 (DOCX 14.0 KB)

Supplementary file3 (PNG  87.0 KB)

High resolution (TIF 321 MB)

Supplementary file4 (PNG 121 KB)

High resolution (TIF 454 MB)

## Data Availability

Data are available from the corresponding author upon reasonable request.
