# Supplementary material for: Off-pump Versus On-pump Coronary Artery Bypass Grafting in Diabetic patients: A Meta-analysis of Observational Studies with a Propensity-Score Analysis
Source: Cardiovasc Drugs Ther. 2024 Jul 11;39(6):1365–74. doi: 10.1007/s10557-024-07603-y (PMC12717115; doi:10.1007/s10557-024-07603-y)
Supplement: Supplementary file 1 — Supplementary file1 (DOCX 16.5 KB) [file 10557_2024_7603_MOESM1_ESM.docx]

APPENDIX E1: DETAILS OF THE FULL SEARCH STRATEGY

Ovid MEDLINE (ALL - 1946 to present)

| Line # | Search |
| --- | --- |
| 1 | **Coronary Artery Bypass/** |
| 2 | (aorta adj2 bypass).tw. |
| 3 | CABG.tw. |
| 4 | (aortic coronary bypass or aorticocoronary anastomosis).tw. |
| 5 | (aorto coronary adj2 (bypass or graft)).tw. |
| 6 | (aortocoronary adj2 (anastomosis or bypass or shunt or graft)).tw. |
| 7 | (coronary adj2 (bypass or graft)).tw. |
| 8 | (Total arterial revascularization or total arterial revascularisation or Multiple arterial revascularization or multiple arterial revascularisation).tw. |
| 9 | or/1-8 |
| 10 | **Diabetes Mellitus/** |
| 11 | (diabetic or diabetes or DM).tw. |
| 12 | or/10-11 |
| 13 | **Coronary Artery Bypass, Off-Pump/** |
| 14 | (off-pump or off pump or beating heart).tw. |
| 15 | or/13-14 |
| 16 | 9 and 12 and 15 |
| 17 | Editorial/ or Letter/ or Comment/ or Meeting abstract/ |
| 18 | (conference paper or conference proceeding* or conference abstract* or meeting abstract or editorial or letter or comment).pt. |
| 19 | 17 or 18 |
| 20 | 16 not 19 |
| 21 | limit 20 to english language |

Searched on December 17, 2023

Embase

Searched on December 17, 2023

| Line # | Search |
| --- | --- |
| 1 | **'coronary artery bypass graft'/exp** |
| 2 | (aorta NEAR/2 bypass):ti,ab,kw |
| 3 | cabg:ti,ab,kw |
| 4 | 'aortic coronary bypass':ti,ab,kw OR 'aorticocoronary anastomosis':ti,ab,kw |
| 5 | ((aortocoronary NEAR/2 bypass):ti,ab,kw) OR ((aortocoronary NEAR/2 graft):ti,ab,kw) OR ((aortocoronary NEAR/2 anastomosis):ti,ab,kw) OR ((aortocoronary NEAR/2 shunt):ti,ab,kw) |
| 6 | (('aorto coronary' NEAR/2 bypass):ti,ab,kw) OR (('aorto coronary' NEAR/2 graft):ti,ab,kw) |
| 7 | ((coronary NEAR/2 bypass):ti,ab,kw) OR ((coronary NEAR/2 graft):ti,ab,kw) |
| 8 | 'total arterial revascularization':ti,ab,kw OR 'total arterial revascularisation':ti,ab,kw OR 'multiple arterial revascularisation':ti,ab,kw OR 'multiple arterial revascularization':ti,ab,kw |
| 9 | #1 OR #2 OR #3 OR #4 OR #5 OR #6 OR #7 OR #8 |
| 10 | **'diabetes mellitus'/exp** |
| 11 | diabetic:ti,ab,kw OR diabetes:ti,ab,kw OR dm:ti,ab,kw |
| 12 | #10 OR #11 |
| 13 | **'off pump coronary surgery'/exp** |
| 14 | 'off pump':ti,ab,kw OR 'beating heart':ti,ab,kw |
| 15 | #13 OR #14 |
| 16 | #9 AND #12 AND #15 |
| 17 | #16 AND ('article'/it OR 'article in press'/it OR 'preprint'/it) |

Cochrane Library (Wiley)

Searched on December 17, 2023

| Line # | Search |
| --- | --- |
| 1 | **MeSH descriptor: [Coronary Artery Bypass] explode all trees** |
| 2 | (aorta NEAR/2 bypass):ti,ab,kw |
| 3 | (CABG):ti,ab,kw |
| 4 | ((aortic coronary bypass or aorticocoronary anastomosis)):ti,ab,kw |
| 5 | (aorto coronary NEAR/2 (bypass or graft)):ti,ab,kw |
| 6 | (aortocoronary NEAR/2 (anastomosis or bypass or shunt or graft)):ti,ab,kw |
| 7 | ((coronary NEAR/2 (bypass or graft))):ti,ab,kw |
| 8 | ((Total arterial revascularization or total arterial revascularisation or Multiple arterial revascularization or multiple arterial revascularisation)):ti,ab,kw |
| 9 | #1 or #2 or #3 or #4 or #5 or #6 or #7 or #8 |
| 10 | **MeSH descriptor: [Diabetes Mellitus] explode all trees** |
| 11 | (diabetic or diabetes or DM):ti,ab,kw |
| 12 | #10 OR #11 |
| 13 | **MeSH descriptor: [Coronary Artery Bypass, Off-Pump] explode all trees** |
| 14 | ((off pump) or off-pump or beating heart):ti,ab,kw |
| 15 | #13 or #14 |
| 16 | #9 and #12 and #15 |
