# Supplementary material for: Off-pump Versus On-pump Coronary Artery Bypass Grafting in Diabetic patients: A Meta-analysis of Observational Studies with a Propensity-Score Analysis
Source: Cardiovasc Drugs Ther. 2024 Jul 11;39(6):1365–74. doi: 10.1007/s10557-024-07603-y (PMC12717115; doi:10.1007/s10557-024-07603-y)
Supplement: Supplementary file 2 — Supplementary file2 (DOCX 14.0 KB) [file 10557_2024_7603_MOESM2_ESM.docx]

Newcastle-Ottawa Scale for quality assessment of the nonrandomized studies included in our analysis.

| Study | Selection | Comparability | Outcome |
| --- | --- | --- | --- |
| Srinivasan | **** | ** | *** |
| Emmert | **** | ** | ** |
| Renner | **** | ** | *** |
| Singh | **** | ** | *** |
| Huang | **** | ** | *** |
| Benedetto | **** | ** | *** |
| Song | *** | ** | *** |
