# Supplementary figures and images for: Off-pump Versus On-pump Coronary Artery Bypass Grafting in Diabetic patients: A Meta-analysis of Observational Studies with a Propensity-Score Analysis
Source: Cardiovasc Drugs Ther. 2024 Jul 11;39(6):1365–74. doi: 10.1007/s10557-024-07603-y (PMC12717115; doi:10.1007/s10557-024-07603-y)

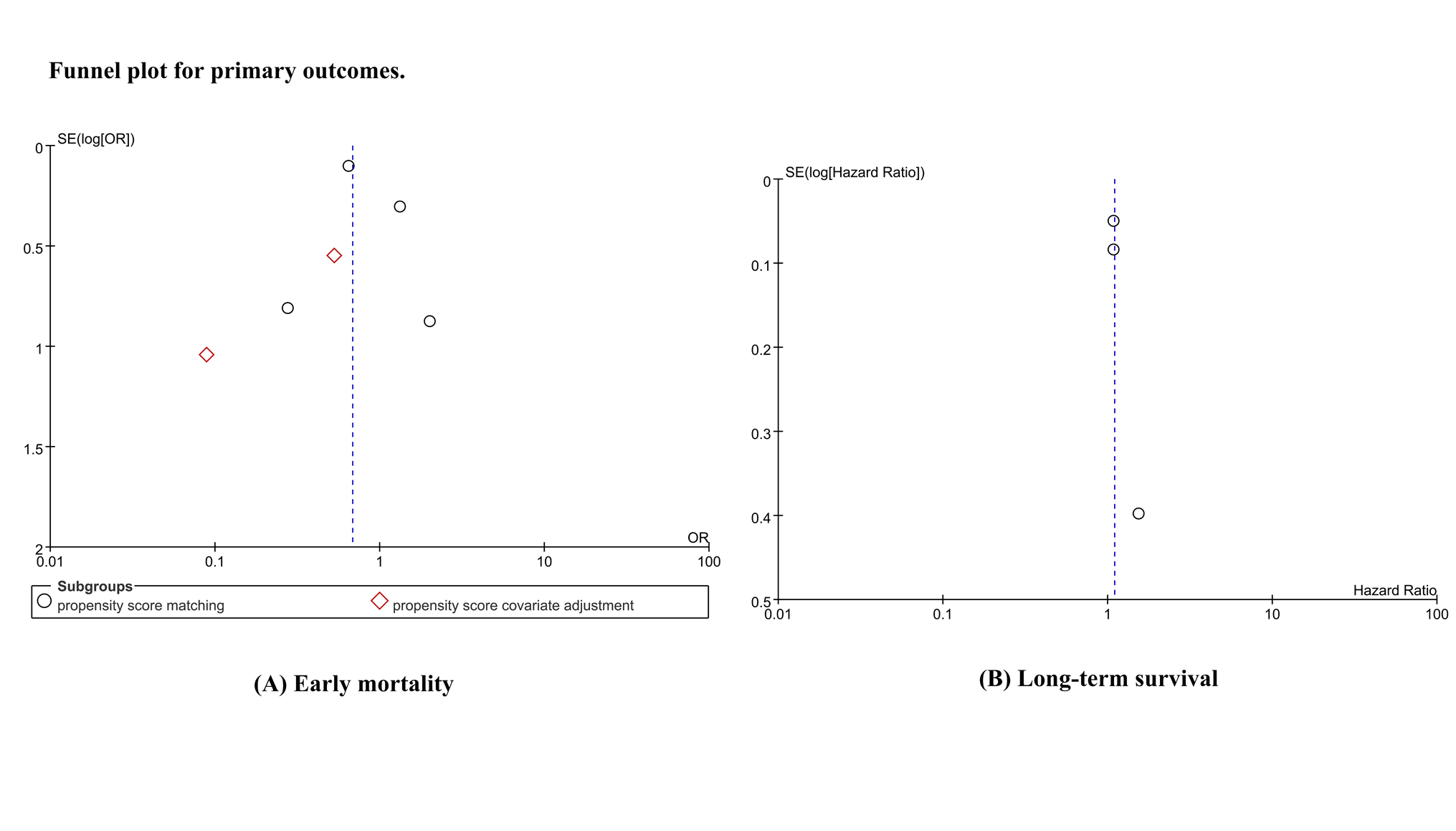

Supplement: Supplementary file 3 — Supplementary file3 (PNG 87.0 KB) [file 10557_2024_7603_Fig5_ESM.png]

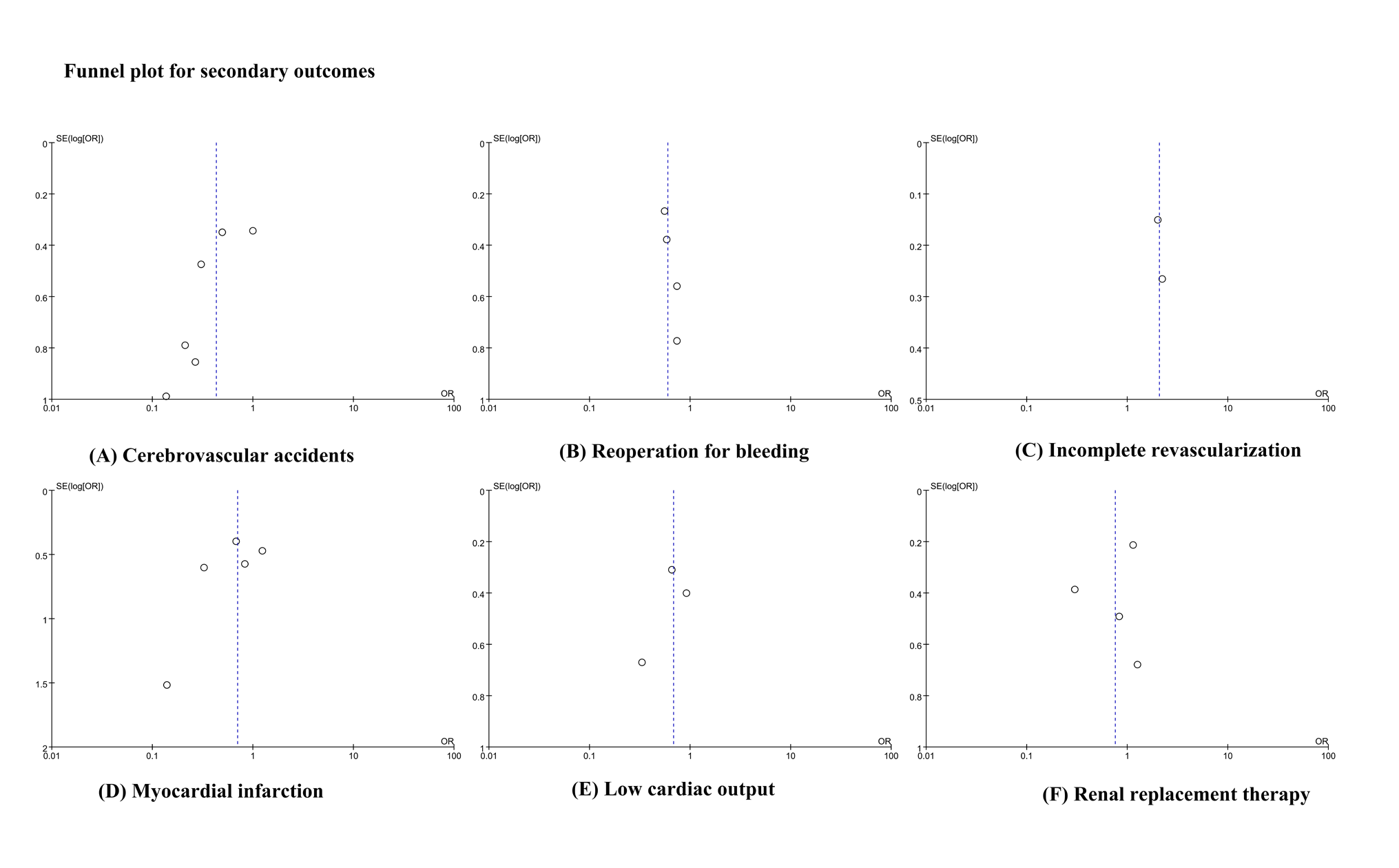

Supplement: Supplementary file 5 — Supplementary file4 (PNG 121 KB) [file 10557_2024_7603_Fig6_ESM.png]
